# Outcomes, Microbiology and Antimicrobial Usage in Pressure Ulcer-Related Pelvic Osteomyelitis: Messages for Clinical Practice

**DOI:** 10.7150/jbji.41779

**Published:** 2020-03-26

**Authors:** Clark D. Russell, Shao-Ting Jerry Tsang, Alasdair Hamish R. W. Simpson, Rebecca K. Sutherland

**Affiliations:** 1 NHS Lothian Infection Service, Regional Infectious Diseases Unit, Western General Hospital, Edinburgh, U.K.; 2 University of Edinburgh Centre for Inflammation Research, Queen's Medical Research Institute, Edinburgh BioQuarter, Edinburgh, U.K.; 3 Department of Orthopaedic Surgery, University of Edinburgh, Chancellor's Building, Edinburgh, U.K.

**Keywords:** osteomyelitis, pressure ulcer, pelvis, spinal cord injuries

## Abstract

**Introduction**: Pressure ulcer-related pelvic osteomyelitis is a relatively under-studied entity in the field of bone infection. We sought to add to the limited evidence base for managing this challenging syndrome.

**Methods**: Cases were identified retrospectively from a surgical database and hospital discharge codes at a U.K. tertiary centre (2009-2018). Risk factors associated with outcomes were analysed by logistic regression.

**Results**: We identified 35 patients (mean age 57.4 years), 69% managed with a combined medical and surgical approach, with mean follow-up of 3.7 years from index admission. Treatment failure (requiring further surgery or intravenous antimicrobials) occurred in 71% and eventual ulcer healing in 36%. One-year mortality was 23%. Lack of formal care support on discharge, post-traumatic (asensate) neurological deficit and index CRP (>184mg/L) were associated with treatment failure (p=0.001). Age (>59.5 years), lack of attempted soft tissue coverage, haemoglobin (<111g/L) and albumin (<25g/L) were associated with non-healing ulcers (p=0.003). Superficial wound swabs had low sensitivity and specificity compared to deep bone microbiology. Infection (based on deep bone microbiology from 46 infection episodes) was usually polymicrobial (87%), commonly involving *S. aureus*, Enterococci, GNB and anaerobes. Antimicrobial duration ranged from 0-103 days (mean 54) and was not associated with subsequent treatment failure.

**Conclusions**: Attempted soft tissue coverage after surgical debridement, ensuring appropriate support for personal care after discharge and nutritional optimisation could improve outcomes. Superficial wound swabs are uninformative and deep bone sampling should be pursued. Long antimicrobial courses do not improve outcomes. Clinicians should engage patients in anticipatory care planning.

## Introduction

Pelvic osteomyelitis occurring as a complication of pressure ulcers results in substantial morbidity and therapeutic challenges, but remains a relatively under-studied entity in the field of bone infection. Such pressure ulcers arise due to immobility, usually in the context of neurological dysfunction 1. Concomitant urinary incontinence can lead to skin irritation further compromising barrier function, and faecal incontinence can lead to wound soiling and introduction of enteric bacteria. Bone sequestra and necrotic connective tissue, devoid of a blood supply, provide a persistent nidus of infection and impair local antimicrobial penetration following systemic administration. Wound closure and healing following surgical intervention are at high risk of failure 2.

A recent systematic review of this challenging syndrome provides an overview of the limited available data pertaining to diagnosis and management 3. Diagnosis itself can be difficult, and contrary to the widely held belief, exposed bone is not necessarily synonymous with histological evidence of osteomyelitis 3. Furthermore, the specificity of CT and MRI has been questioned when histology is considered the gold standard 4-9. The optimum duration of antimicrobial therapy has not been defined, nor the sub-group of patients who will derive benefit from surgical intervention.

Utilising a single centre cohort of patients we sought to add to the limited evidence base for this challenging syndrome. We aimed to ask clinically-relevant questions including what factors are associated with treatment failure and failure of ulcer healing, how do deep and superficial microbiological samples compare, and whether antimicrobial duration is associated with outcome?

## Methods

### Data collection

Patients from a U.K. tertiary centre were identified from a local database of patients undergoing surgical management of osteomyelitis, supplemented by an electronic search of the electronic patient record (TrakCare®) for episodes with diagnoses coded (Table S1) as osteomyelitis of the pelvis, sacrum, coccyx or ischium, or infected pressure ulcers (December 2009-December 2018). Cases identified by the latter search were then reviewed to determine if they met the case definition (below). The case log was created following a search of the pan-regional electronic perioperative management system (Operating Room Scheduling and Office System), reflecting an entire regional surgical practice. TrakCare® is a region-wide electronic medical record system. It collects the medical data from patient interactions in all secondary and tertiary centres throughout the region. Death is recorded on TrakCare® and linked to national death records by the patient's community health index number, a unique patient identifier assigned to all users of the healthcare system in Scotland. Chart review was undertaken and relevant clinical, radiological and microbiological details were recorded. The project was reviewed and approved by the NHS Lothian Musculoskeletal- Orthopaedic Quality Improvement Team.

### Definitions

Cases were defined as adults (≥18 years) requiring inpatient management of clinically and radiologically diagnosed pressure ulcer-related pelvic osteomyelitis. Patients were excluded if they presented with soft tissue infection involving an ulcer or visible bone (i.e. grade 4 ulcer) without evidence of underlying osteomyelitis. The index episode was defined as the initial presentation requiring inpatient management including the first surgical procedure. Treatment failure was defined as: (i) further surgical debridement, (ii) further positive bone cultures, or (iii) re-admission for intravenous antimicrobials for treatment of pelvic osteomyelitis. This expands on a recently used definition with the addition of requirement for further antimicrobials, which we contend is a relevant outcome for patients 10. Patients requiring surgical debridement underwent wide resection with dissection down to bleeding bone. Bone prominences were removed to prevent future skin pressure. Where possible, defects were closed primarily or covered with a flap reconstruction. If neither option was possible in the first instance, negative pressure wound dressings were applied to facilitate delayed closure +/- soft tissue reconstruction. Multiple intraoperative deep bone samples were taken for microbiological investigation (usually six) and at least one sample for histological analysis (soft tissue +/- bone).

### Significant microbiology

Isolates of uncertain clinical significance in the context of osteomyelitis (*e.g.* Corynebacterium spp., coagulase-negative Staphylococci [excluding *S. lugdunensis*], viridans group Streptococci, fungi) were considered as significant if they were recovered from >1 deep sample from the same procedure and had a similar antibiogram, or if they were recovered from the same patient during a subsequent infection episode.

### Statistical analysis

Fisher's exact-test (or a Chi-square test where appropriate) was used to analyse the difference between groups for categorical variables, and a univariate analysis of variance (or Mann Witney U test for non-parametric data) for continuous variables. A Kaplan Meier survival curve with a log-rank analysis was used for comparative evaluations of treatment failure and mortality in time. Possible risk factors for treatment failure were selected and analysed using univariate analysis as detailed above. Receiver operator characteristic (ROC) analysis was performed to quantitate the predictive performance of risk factors identified on univariate analysis, in addition the Youden's J statistic was calculated for identified continuous variables. Identified variables on univariate analysis were assessed for multicollinearity in a binomial logistic regression analysis with only statistically significant variables included in the risk score model. All analyses were two-tailed and p-values <0.05 were considered to be statistically significant. Statistical analysis was performed using SPSS, version 24.0 (SPSS Inc. Chicago, IL) and Prism, version 8.0 (GraphPad Software Inc. San Diego, CA).

## Results

### Patient characteristics

Thirty five patients with pressure ulcer-related pelvic osteomyelitis were included. The mean age was 57.4 years (95% CI 52.1-62.8) and 26/35 (74%) were male. Most patients (32/35) had neurological dysfunction resulting in immobility, with frailty (2/35) and rheumatoid arthritis (1/35) as predisposing factors in the remaining cases. Other co-morbidities were uncommon (median 0, range 1-3; Table 1). The most common site of osteomyelitis was the ischium (18/35). Full patient characteristics are reported in Table 1. 31/35 index admissions were directly due to pelvic osteomyelitis (due to other intercurrent infections in the remaining 4) and in 27 such instances the patient was acutely unwell due to osteomyelitis and/or skin and soft tissue infection (SSTI) involving the ulcer (admitted electively for surgery in remaining 4). The responsible pressure ulcers had been present for a median of 4 months (IQR 1.75-12.0) and were most commonly grade four (21/35) or three (13/35). There was one case of squamous cell carcinoma as a complication of the infection.

Diagnostic parameters at index presentation are presented in Table 1. Clinical signs of SSTI involving the ulcer were present in 29/35 cases. Samples of bone were sent to histopathology in nine cases, with active inflammation identified in seven. In the two cases where only reactive changes were reported, MRI identified sequestra and erosions, and bone samples from the same operations sent to microbiology were culture-positive.

### Management

The majority of patients were managed with a combined medical and surgical approach (24/35). One patient underwent flap reconstruction during their index presentation followed by one re-admission for debridement and flap elevation. Three patients underwent flap reconstruction on their second re-admission and required no further intervention. Of the patients who underwent direct primary closure, 11/14 experienced wound breakdown with six requiring further surgical intervention and five requiring further antimicrobials only. They required a median of one further admission (range 0-7), with 7/14 having wounds that had healed at the time of chart review.

**Figure 1:**
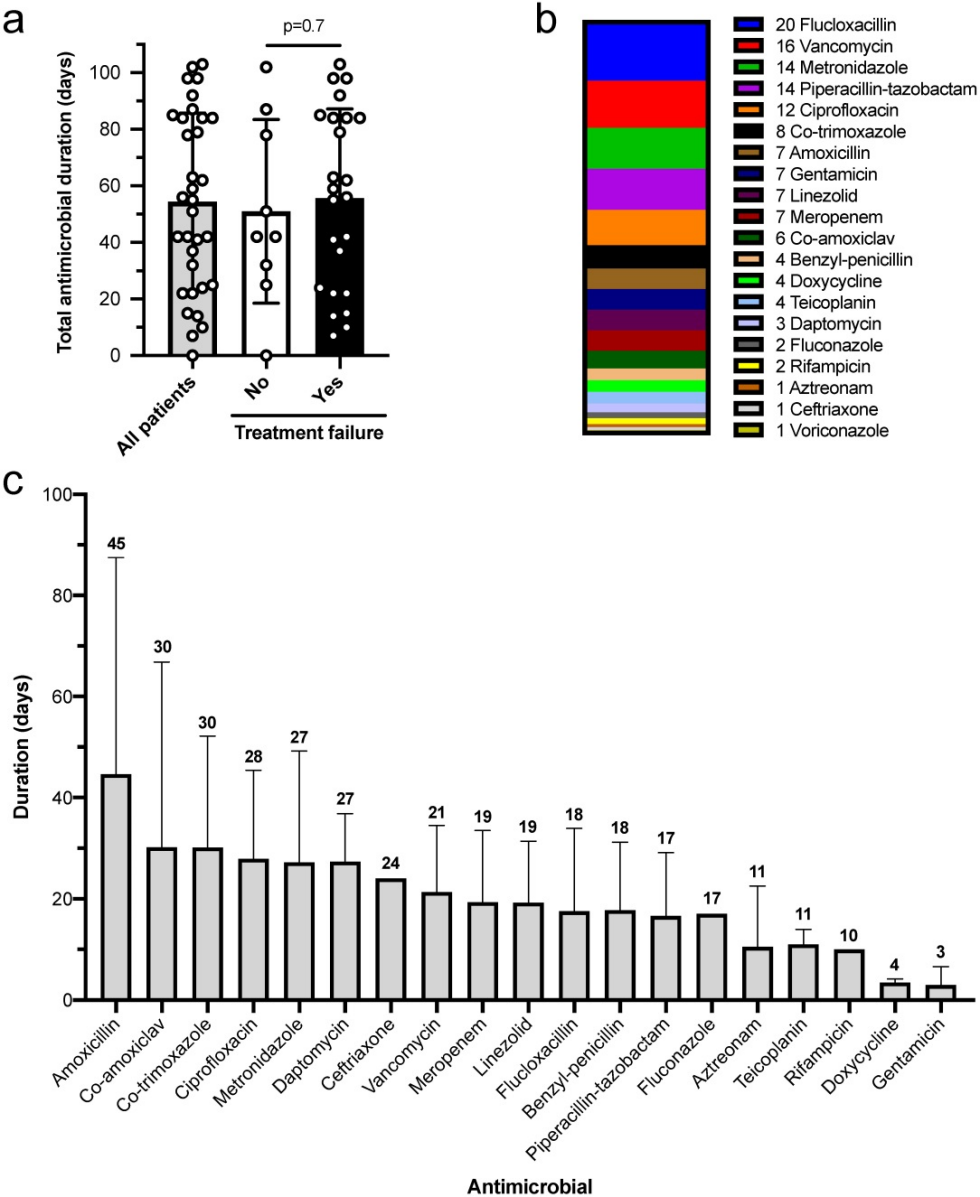
** Antimicrobial usage. a)** The total antimicrobial duration at index presentation was not associated with subsequent treatment failure (groups compared by unpaired t test). **b)** Antimicrobial agents used during management of index episode. **c)** Duration of each antimicrobial agent during index episodes (number is mean). Antimicrobial data available for 33 patients. Graphs show mean and standard deviation.

The mean total antimicrobial duration during the index episode was 54 days (standard deviation ±31 days), but this was highly variable (Figure 1A), with a range of 0-103 days. There was no association between antimicrobial duration and subsequent treatment failure (51.0 days treatment success, 55.7 days treatment failure, p=0.7). When patients were stratified into receiving ≤6 weeks or >6 weeks of antimicrobials there remained no association with subsequent treatment failure (p=0.7). Patients received a median of 4 antimicrobials in total (IQR 2-6), with flucloxacillin, vancomycin, metronidazole, piperacillin-tazobactam and ciprofloxacin constituting over half (Figure 1B). Broad spectrum beta-lactams (piperacillin-tazobactam, co-amoxiclav, meropenem) were used for average durations of 17, 30 and 19 days respectively (Figure 1C). Despite the extensive antimicrobial exposure there was only one case of *Clostridioides difficile* infection. Seven patients developed adverse drug reactions involving antimicrobials, including nephrotoxicity (vancomycin and gentamicin), hyper-sensitivity (teicoplanin and co-trimoxazole) and anaemia (linezolid).

### Outcomes

#### Follow-up and mortality

**Figure 2:**
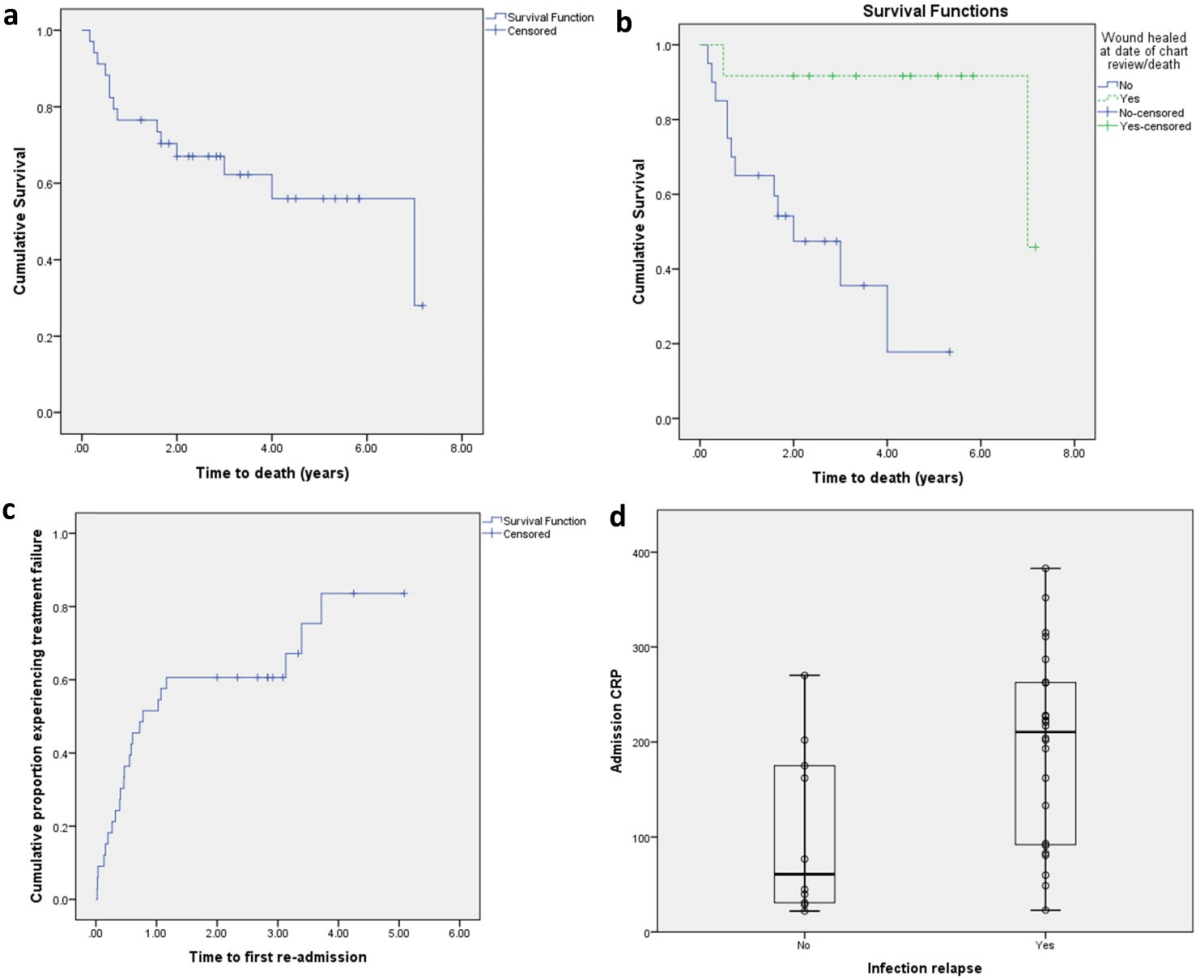
** Clinical outcomes. a)** Kaplan-Meier analysis of patient survival. **b)** Log rank analysis of patient survival between those with healed and non-healed pressure ulcers at time of final follow-up.** c)** Treatment failure. **d)** Admission C-reactive protein (mg L^-1^) at index presentation in the prediction of treatment failure.

At time of chart review 15/35 (43%) patients had died, with 8/35 (23%) dying within twelve months of their index episode. Kaplan-Meier analysis provided an estimated median time to death from index episode of 7.0 years (95% CI 2.7-11.3) for the entire cohort (Figure 2A). Median time to death in patients with healed pressure ulcers was 7.0 years (95% CI not available) and unhealed pressure ulcers was 2.0 years (95% CI 0.6-3.4 years; p=0.003, Figure 2B). Excluding the 15 deaths, there was a mean follow-up of 3.7 years (range 1.3-7.2).

#### Ulcer healing

Unhealed pressure ulcers were present in 21/33 (64%) patients at the time of chart review or death. Univariate analysis identified older age (mean 63.4 vs. 48.9 years, p=0.010), diagnosis of diabetes mellitus (7/21 vs. 0/12, p=0.032), lower haemoglobin and albumin at index presentation (mean 105 vs. 125g L^-1^, p=0.007; and 22.1 vs. 27.2g L^-1^, p=0.040, respectively), and not attempting soft tissue coverage at index presentation (8/20 vs. 10/12 primary closure as opposed to no closure or negative pressure wound dressing, p=0.028) to be associated with an unhealed ulcer. Receiver operating characteristic (ROC) analysis is presented in Table 2. Binomial logistic regression analysis (X^2^(4) = 15.781, p=0.003) found that admission haemoglobin (<111g L^-1^), admission albumin (<25g L^-1^), lack of soft tissue coverage, and patient age at index presentation (>59.5 years) accounted for 79.3% (Nagelkerke R^2^) of the variance in the failure of pressure ulcers to heal and correctly classified 90.5% of cases (Table S2).

#### Treatment failure

Treatment failure occurred in 25/35 (71.4%) cases. The median time from index presentation was 9.3 months (95% CI 2.7-15.9) (Figure 2C). Univariate analysis found that lack of formal care support on discharge (relative risk ratio [RRR] 3.2, p=0.027), an underlying asensate post-traumatic neurological deficit (RRR N/A, p=0.030), and higher index admission C-reactive protein (194 vs. 105mg L^-1^, p=0.021) (Figure 2D) were associated with treatment failure. Bacteraemia on index admission was not associated with subsequent treatment failure (p=1.0). Receiver operating characteristic (ROC) analysis is presented in Table 2. Binomial logistic regression analysis (X^2^(3) = 15.588, p=0.001) found that index CRP (>184mg L^-1^), lack of formal care support and a post-traumatic neurological deficit accounted for 52.4% (Nagelkerke R^2^) of the variance in treatment failure and correctly classified 82% of cases (Table S2).

### Microbiology

Considering each index admission and any subsequent treatment failures as an infection episode, positive microbiology was available for 80/90 episodes, with growth from a deep bone sample in 46 instances, blood culture in eleven, and a superficial wound swab alone in 26. Bacteraemia was due to *S. aureus* (4 MSSA, 1 MRSA), *S. agalactiae* (n=2), *S. dysgalactiae* (n=1), *E. faecium* (n=1) and Gram negative bacilli (GNB; n=3; Table S3). Deep bone samples were obtained intra-operatively in 43/46 episodes and by a Radiologist in 3 medically-managed episodes. In 13/18 cases with sufficient data, antimicrobials were administered prior to surgery. Significant microbiology from deep bone samples is shown in Table S4. Infection was usually polymicrobial (40/46) and microbiologically heterogeneous, with *S. aureus*, Enterococci, GNB and anaerobes the most frequent pathogens. Gram positive bacteria were involved in 43/46, Gram negative in 26/46 and anaerobes in 16/46 (Figure S1). The sensitivity and specificity of superficial wound swabs for detection of *S. aureus*, Enterococci, GNB and anaerobes were calculated (Table 3), demonstrating inadequate performance when compared to deep bone samples from the same infection episode. In particular, the specificity of detection of *S. aureus* (36.4%) and GNB (43.8%) were particularly low. The sensitivity for detecting individual organisms from superficial swabs was also low: 47.4% for GNB, 50.0% for Enterococci, 55.6% for anaerobes and 76.5% for *S. aureus*. Overall, 45/91 isolates (49.5%) from deep bone samples were detected on superficial swabs in episodes where both were obtained. 10/35 patients were colonised with MRSA and five developed MRSA infection.

Sequential intra-operative bone samples were available for 12 patients (median 2 infection episodes/patient, range 2-4) encompassing a total of 30 infection episodes a median of 84 days apart (IQR 23-371). All were polymicrobial. Considering the combined 101 isolates from these episodes, 44 (43.6%) were involved in subsequent infections. Considering individual patients, in 9/12 individuals at least one organism was involved in a further infection episode.

## Discussion

Pressure ulcer-related pelvic osteomyelitis is a complex infection but remains relatively under- studied. In this retrospective cohort study of 35 patients we sought to ask clinically relevant questions. This study is limited by its small sample size, single-centre design, retrospective nature and an inability to determine the clinical characteristics determining decisions to manage patients with or without surgery (including patient preference to undergo surgery). If a patient relocated to outwith the study region then instances of treatment failure could have been missed by the case ascertainment strategy.

### Host nutritional status

Anaemia and hypo-albuminaemia were identified as risk factors for a non-healing ulcer and could relate to host nutritional status in the context of a catabolic state induced by chronic inflammation. Pre-operative anaemia is associated with an increased risk of post-operative infections in patients undergoing orthopaedic surgery 11-13, with the effect thought to be associated with an increased requirement for post-operative blood transfusion 12-15. Thus, there is a risk-benefit balance between transfusion-related immune modulation and optimising oxygenation of the hypo-perfused wound. Measures such as nutritional optimisation and haematinic assessment should be prioritised to reduce reliance on blood transfusion. It has been suggested that blood transfusion should be avoided intra- operatively 16 and, if anticipated, should be administered at least 48 hours before surgery.

Malnutrition is associated with multiple adverse outcomes following orthopaedic surgery: prolonged length of in-patient stay 17, delayed wound healing 18, surgical site infection (SSI) 19, sepsis 20, and death 20. Serum albumin has been reported as a marker of nutritional status and hypo-albuminaemia (<35g L^-1^) has consistently been associated with increased risk of SSI following orthopaedic surgery 17, 20-23. A recent international consensus meeting on orthopaedic infections strongly agreed that malnutrition was a modifiable risk factor for delayed wound healing 24 and SSI 25. A possible alternative explanation for the observed association with hypo-albuminaemia and ulcer healing is loss of albumin *via* wound exudate, in a process similar to that occurring in burns patients 26.

### Wound coverage

Attempted soft tissue coverage was associated with eventual ulcer healing in this cohort. Two centres have reported good long-term outcomes using myo-cutaneous flaps. A flap was constructed in 157/157 cases in one cohort (all pressure ulcers; 108 with underlying osteomyelitis) and 30/61 in another (all pelvic osteomyelitis; 41 with pressure ulcers) 10, 27. In this latter cohort, 5/7 recurrences occurred in patients with osteomyelitis due to pressure ulcers. One retrospective study specifically sought risk factors for ulcer recurrence after debridement, reporting a hazard ratio of 0.8 for flap use, but with a wide 95% confidence interval of 0.2 to 3.1 28. However, flaps were only constructed in 25/70 patients and underlying osteomyelitis was absent in 18/70 lesions. Another centre reported flap construction after debridement in only 7/55 cases but no specific associated outcomes 29.

### Predicting and preventing treatment failure

Treatment failure was predicted by admission CRP, lack of formal care support after discharge and a post-traumatic neurological deficit. CRP concentration at presentation has previously been found to independently predict treatment failure in musculoskeletal infections, possibly related to the burden of infection 30, 31.

In the context of preventing treatment failure, a holistic perspective is warranted, considering patient concordance with treatment, wound care and pressure off-loading. Any underlying psychosocial factors that contributed to initial ulcer development should be identified and addressed, including formal care support after hospital discharge 10, 32. Whilst Tissue Viability nursing input is the standard of care for patients in hospital, it is not known what wound care was delivered to patients in this cohort in the community after discharge.

A multi-disciplinary team approach to musculoskeletal infection links clinicians (infection specialists, musculoskeletal radiologists, anaesthetists, plastic surgeons and orthopaedic surgeons) and allied health and care professionals (nurses, dieticians, physiotherapists, occupational therapists, pharmacists, psychologists and social care co-ordinators) 33. The cohort study from Dudareva and colleagues from the Oxford Bone Infection Unit reported a low recurrence rate (11%) in the setting of an established multi-disciplinary approach to pelvic osteomyelitis 10. The definition of treatment failure used in this study did not include re-admission for intravenous antimicrobials which we did include, and not all patients had pressure ulcer-related osteomyelitis. Therefore, outcomes are not directly comparable between our cohort and theirs.

### Microbiological sampling and aetiology

Based on the results of deep bone cultures we report a microbial aetiology comparable to other investigators 6, 7, 10, 29. Importantly, we show superficial wound swabs are poor predictors of deep bone microbiology, thus are uninformative in determining aetiology of underlying osteomyelitis. One series reported that the implicated pathogen identified during recurrent episodes was unrelated to the index isolate in 86% of cases, and our data similarly show that only 43.6% of recovered organisms were identified in subsequent infections 28. Therefore, prior microbiology results do not reliably predict the aetiology of subsequent infection episodes and there is evidence for microbiological recurrence and *de novo* re-infection occurring simultaneously.

### Antimicrobial management

We identified significant heterogeneity in the total duration of antimicrobial therapy during the index admission but no association with subsequent treatment failure. To our knowledge this is the first report of this finding in a cohort of patients exclusively with pressure ulcer-related pelvic osteomyelitis, and supports similar findings by other investigators who have demonstrated that antimicrobial duration is not associated with healing of infected sacral pressure ulcers (with or without underlying osteomyelitis) 6, 34, 35. The recent OVIVA trial included 6.8% patients with spinal/pelvic osteomyelitis but overall 322/376 patients with chronic osteomyelitis (any site) had undergone debridement, therefore to extrapolate the trial findings to pressure ulcer-related pelvic osteomyelitis, further work will be required to define the sub-group of patients requiring surgical intervention 36. The large cohort study from Bodavula and colleagues reports that combined medical and surgical management is associated with less re-admissions in the following year compared to antimicrobials alone 29. Whilst this is an informative finding, the chronicity of this infection dictates that analysis of more than one year of follow-up will be required to reach definitive conclusions. Finally, the extent of osteomyelitis is likely to be a relevant factor when determining the length of antimicrobial therapy required, for example superficial cortical versus medullary involvement. There was insufficient histological information available to comment on this in our cohort.

### Anticipatory care planning

In this series, 43% of patients had died after mean follow-up of 3.7 years. Other investigators have reported 20-25% mortality with mean follow-up of 2.8-4.7 years 10, 37. These findings speak to the importance of anticipatory care planning and 'Realistic Medicine' following an index admission with pressure-ulcer related pelvic osteomyelitis 38. Clinicians must continuously evaluate the appropriateness of aggressive management, entailing deep bone sampling, surgical debridement and embarking on lengthy and complex antimicrobial regimens. Such measures should not be pursued if and when a time arises when any benefits derived are outweighed by deleterious effects on the patient's overall physiological, psychological and social wellbeing, viewed from the patient's perspective. This is particularly pertinent when considering the high treatment failure rate resulting in re-admission and underscores the importance of preventing this. Realistic goals of care could include promoting healing of existing ulcers, preventing additional ulcers and treating acute flares of SSTI involving the ulcer with brief courses of antimicrobials.

## Conclusions

Identification of the sub-group of patients who will benefit from surgery remains an important goal to advance the management of this disease. Formal personal care and nutrition are potentially modifiable factors associated with outcomes and should feature in strategies to optimise surgical outcomes. Admission CRP could be used to identify patients at risk of poor outcomes who may need closer follow- up. For patients undergoing surgical intervention, this should be performed as a combined ortho-plastic procedure to facilitate adequate debridement and early soft tissue reconstruction, when primary closure is not possible 10, 27. Microbiological evaluation should involve deep bone sampling and not rely on superficial wound swabs. Antimicrobial duration is not associated with risk of treatment failure and shorter courses may be appropriate in some cases. The role of antimicrobials may be secondary to optimising the bio-psycho-social factors required for successful surgical intervention and subsequent wound healing. Almost one quarter of patients died within one-year of their index episode and therefore anticipatory care planning should be considered. A holistic approach to management is required, including dietetics, careful discharge planning, social care, tissue viability, infection specialists, plastic surgeons and orthopaedic surgeons.

## Supplementary Material

Supplementary tables.

## Figures and Tables

**Table 1:** Characteristics of included patients with pressure ulcer-related pelvic osteomyelitis (n = 35)

Characteristics	N (%)
**Male (%)**	26 (74)
**Age, mean (95% CI) years**	57.4 (52.1-62.8)
**Neurological dysfunction**	32 (91.4)
Hemiplegia	2 (5.7)
Paraplegia	27 (77.1)
Quadriplegia	3 (8.6)
**Aetiology of neurologic dysfunction**	
Trauma	10 (28.6)
Multiple sclerosis	8 (22.9)
Congential	7 (20.0)
Other^a^	7 (20.0)
**Defunctioning colostomy**	4
**Long-term urinary catheter**	24
**Cierny-Mader host classification**	
A	8 (22.9)
B	26 (74.3)
C	1 (2.9)
**Co-morbidities**	
Diabetes mellitus	8 (22.9)
Ischaemic heart disease or heart failure	6 (17.1)
Chronic lung disease	4 (11.4)
Other^b^	4 (11.4)
**Site of pressure ulcer**	
Ischium	19
Sacrum	13
Femur	3
**Site of osteomyelitis**	
Ischium	18 (51.4)
Proximal femur	5 (14.3)
Sacro-coccygeal	5 (14.3)
Sacrum	3 (8.6)
Ischium and pubic ramus	2 (5.7)
Ischium and proximal femur	1 (2.9)
Coccyx	1 (2.9)
**Diagnostic parameters at index admission**	
Total white cell count, x10^9^ L^-1^ (mean, 95% CI)	12.7 (10.6-14.0)
Neutrophil count, x10^9^ L^-1^ (mean, 95% CI)	10.2 (8.3-12.2)
C-reactive protein, mg L^-1^ (mean, 95% CI)	168 (132-205)
**Radiological findings (n=33)^c^**	
Bone marrow oedema	25 (75.8)
Bone cortex erosion	19 (57.6)
Sequestra	5 (15.2)
Myositis	20 (60.6)
Collection or abscess	15 (45.5)
**Histopathological findings (n=9)**	
Acute (neutrophilic) inflammation	1 (11.1)
Chronic (monocytic) inflammation	4 (44.4)
Acute and chronic inflammation	2 (22.2)
Reactive changes only	2 (22.2)
**Significant microbiology from deep bone sample (n=25)**	25 (100.0)
**Surgical intervention**	24 (68.6)
Debridement of tissue	2 (9.1)^d^
Debridement of tissue and bone	20 (90.9)^d^
Soft tissue coverage (direct primary closure or flap)	18 (81.8)^d^
**Outcomes**	
Treatment failure	25 (71.4)
Ulcer healing^e^	12 (36.4)^e^
1-year mortality^f^	8 (22.9)

**a** encephalitis (n=3), subarachnoid haemorrhage (n=1), stroke (n=1), cauda equina syndrome (n=1), spinal cord infarct (n=1). **b** Other: chronic kidney disease (n=2); chronic liver disease (n=1); active malignancy (n=1). **c** radiology results available for 33/35 patients: 26 MRI, 7 CT.**d** data available for 22 patients.**e** at time of chart review, data available for 33 patients. **f** relative to date of index admission.

**Table 2:** Receiver operator characteristic analysis of risk factors associated with outcomes

	Threshold^a^	Sensitivity (%)	Specificity (%)	AUC	Rating	p-value	AUC 95% CI
**Ulcer healing**							
Age at index admission	59.5 years	70	75	0.75	Fair	0.019	0.57-0.93
Diabetes mellitus	NA			0.66	Poor	0.144	0.47-0.85
Admission haemoglobin	111g L^-1^	100	75	0.81	Good	0.039	0.63-0.99
Admission albumin	25g L^-1^	80	81	0.84	Good	0.026	0.66-1.00
Soft tissue coverage after debridement	NA			0.73	Fair	0.032	0.55-0.92
**Treatment failure**							
No formal care support	NA			0.72	Fair	0.045	0.53-0.91
Post-traumatic neurological deficit	NA			0.70	Fair	0.068	0.53-0.87
Admission C-reactive protein	184mg L^-1^	63	80	0.78	Fair	0.013	0.60-0.95

**a** Threshold for optimal diagnostic accuracy derived by calculation of the Youden J statistic. NA: not applicable (categorical variable); AUC: area under the curve

**Table 3:** Sensitivity and specificity of superficial wound swabs compared to deep bone samples

	Sensitivity (95% CI)	Specificity (95% CI)
*Staphylococcus aureus*	76.5 (50.1-93.2)	36.4 (10.9-69.2)
Enterococci	50.0 (21.1-78.9)	86.7 (59.5-98.3)
Gram negative bacilli	47.4 (24.5-71.1)	43.8 (19.8-70.1)
Anaerobes	55.6 (21.2-86.3)	84.2 (60.4-96.6)
