# Supplementary material for: Outcomes, Microbiology and Antimicrobial Usage in Pressure Ulcer-Related Pelvic Osteomyelitis: Messages for Clinical Practice
Source: J Bone Jt Infect. 2020 Mar 26;5(2):67–75. doi: 10.7150/jbji.41779 (PMC7242403; doi:10.7150/jbji.41779)
Supplement: Supplementary file 1 — Supplementary tables. [file jbjiv05p0067s1.pdf]

SUPPLEMENTARY DATA

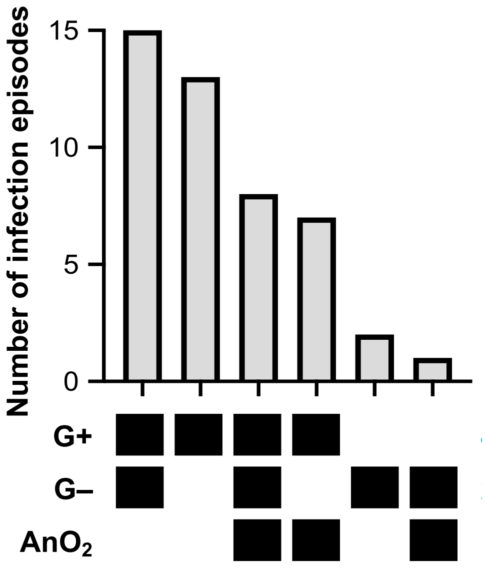

Figure S1: Bacteria recovered from deep bone samples
Infection was usually polymicrobial (40/46 episodes).
G+: Gram positive; G–: Gram negative; AnO_2: anaerobe.

Table S1: ICD-10 codes used in electronic patient record search

Diagnosis	ICD-10 code
Osteomyelitis of the pelvis, sacrum, coccyx or ischium	M86.95
Stage I decubitus ulcer and pressure area	L89.0
Stage II decubitus ulcer	L89.1
Stage III decubitus ulcer	L89.2
Stage IV decubitus ulcer	L89.3
Decubitus ulcer and pressure area, unspecified (Decubitus [pressure] ulcer without mention of stage)	L89.9

Table S2: Model characteristics for predicting outcomes

Characteristic	Value
**Ulcer healing**	
Sensitivity	80%
Specificity	93.8%
Positive predictive value	80%
Score = 1	44%
Score = 2	50%
Score = 3	100%
Score = 4	100%
Negative predictive value	94%
**Treatment failure**	
Sensitivity	92%
Specificity	60%
Positive predictive value	85%
Score = 1	57%
Score = 2	83%
Score = 3	100%
Negative predictive value	75%

Table S3: Bacteraemia

Organism	N[a]	Present in deep bone sample[b]
**Gram positive**		
S. aureus	5	NA
MSSA	4	
MRSA	1	
S. agalactiae	2	1/1
S. dysgalactiae	1	NA
E. faecium	1	NA
**Gram negative**	3	
P. mirabilis	1	0/1
K. pneumoniae	1	1/1
B. ovatus	1	NA

[a]bacteraemia was polymicrobial in 1 case (MSSA and *S. agalactiae*).
[b]denominator is number with deep tissue sample from same infection episode.
NA: not applicable (i.e. no deep bone sample from same infection episode).

Table S4: Microbial aetiology determined by deep bone samples

Organism	N infection episodes (deep bone samples)	Recovered from concurrent wound swab[a]
**Gram positive**		
Staphylococcus aureus	21	13/17
Meticillin-resistant	4	3/3
Enterococcus faecalis	18	5/10
Enterococcus faecium	7	1/3
Vancomycin-resistant	5	1/1
Staphylococcus epidermidis	6	1/3
Streptococcus agalactiae	5	2/2
Streptococcus anginosus	4	0/3
Staphylococcus lugdunensis	4	0/3
Corynebacterium striatum	4	0/1
Group G Streptococcus	3	3/3
Streptococcus dysgalactiae	2	–
Corynebacterium amycolatum	2	0/2
Pedicoccus spp.	2	0/1
Staphylococcus capitis	2	
α-haemolytic Streptococci, not speciated	1	–
Streptococcus pyogenes	1	0/1
Actinomyces turicensis	1	0/1
Gemella morbillorum	1	0/1
Propionibacterium avidum	1	–
Streptococcus oralis	1	0/1
Streptococcus sanguinis	1	–
**Gram negative**		
Escherichia coli	15	4/10
Klebsiella pneumoniae	6	1/3
Pseudomonas aeruginosa	6	2/3
Proteus mirabilis	5	2/3
Enterobacter cloacae	1	–
Morganella morganii	1	–
Prevotella bivia	1	–
Providencia stuartii	1	0/1
Proteus vulgaris	1	–
**Anaerobic**		
Bacteroides fragilis	4	0/3
Anaerobes, not speciated	4	1/1
Fingoldia magna	3	1/2
Peptoniphilus harei	3	1/2
Peptostreptococcus anaerobius	2	1/1
Bacteroides thetaiotamicron	2	0/1
Clostridium perfringens	1	1/1
Clostridium innocuum	1	1/1
Clostridium spp.	1	1/1
Eggerthella lenta	1	0/1
Fusobacterium gonidiaformans	1	–
**Fungi**		
Candida albicans	2	–
Candida glabrata	1	0/1

[a] denominator is number of episodes with superficial wound swab and deep bone sample obtained concurrently.
